# Transcriptome and Lipid Metabolomics-Based Discovery: Glycyrrhizic Acid Alleviates *Tripterygium* Glycoside Tablet-Induced Acute Liver Injury by Regulating the Activities of CYP and the Metabolism of Phosphoglycerides

**DOI:** 10.3389/fphar.2021.822154

**Published:** 2022-02-14

**Authors:** Qiaoli Shi, Qixin Wang, Jiayun Chen, Fei Xia, Chong Qiu, Min Li, Minghong Zhao, Qian Zhang, Piao Luo, Tianming Lu, Ying Zhang, Liting Xu, Xueling He, Tianyu Zhong, Na Lin, Qiuyan Guo

**Affiliations:** ^1^ Artemisinin Research Center and Institute of Chinese Materia Medica, China Academy of Chinese Medical Sciences, Beijing, China; ^2^ China Academy of Chinese Medical Sciences, Beijing, China; ^3^ Key Laboratory of Prevention and Treatment of Cardiovascular and Cerebrovascular Diseases, Ministry of Education, Gannan Medical University, Ganzhou, China; ^4^ Laboratory Medicine, First Affiliated Hospital of Gannan Medical University, Ganzhou, China

**Keywords:** liver injury, glycyrrhizic acid, tripterygium glycoside tablet, lipid metabolomics, transcriptome

## Abstract

**Background:** Glycyrrhizic acid (GA) has been reported to be liver protective; however, the characters and underlying mechanisms of GA against tripterygium glycoside tablet (TGT)-induced acute liver injury remain unelucidated.

**Hypothesis/Purpose:** We assumed that GA could relieve TGT-induced acute liver injury by regulating liver function-related genes and lipid metabolites.

**Study Design:** TGT-induced acute liver injury models were constructed *in vivo* and *in vitro*. Then the liver protective effect and mechanisms of GA were investigated by a combination of transcriptome, lipid metabolomics, and experimental validation.

**Methods:** Intraperitoneal injection of GA was given in advance for six successive days. Then, the TGT-induced acute liver injury model was constructed by a single oral administration of TGT at 270 mg/kg, except for the normal group. All animals were sacrificed 18 h later. The serum levels of aspartate aminotransferase (AST), alanine aminotransferase (ALT), alkaline phosphatase (ALP), total bilirubin (TBIL), glutathione peroxidase (GSH-PX), and superoxide dismutase (SOD) were quantified. Liver tissues were used to observe pathological changes through hematoxylin–eosin (HE) staining and selected for transcriptome and metabolome sequencing. The underlying mechanisms were analyzed and further validated both *in vivo* and *in vitro*.

**Results:** Pre-administration of GA markedly decreased the serum concentrations of AST, ALT, ALP, and TBIL but increased those of SOD and GSH-Px, improving the liver morphology of mice with TGT-induced acute liver injury. In addition, GA significantly increased the gene levels of Cyp2b13, Cyp2c69, Cyp3a16, Cyp3a44, Fmo3, and Nipal1. Differentially accumulated metabolites were screened and classified as phosphatidylcholine (PC) and phosphatidylethanolamine (PE). The *in vitro* results indicated that pre-administration of GA markedly alleviated the inhibitory effect of TGT on BRL-3A activity.

**Conclusion:** This study combined transcriptome, lipid metabolomics, and experimental validation to offer convincing evidence that GA alleviates TGT-induced acute liver injury partially by regulating the activities of CYP and the metabolism of PC and PE.

**GRAPHICAL ABSTRACT:**
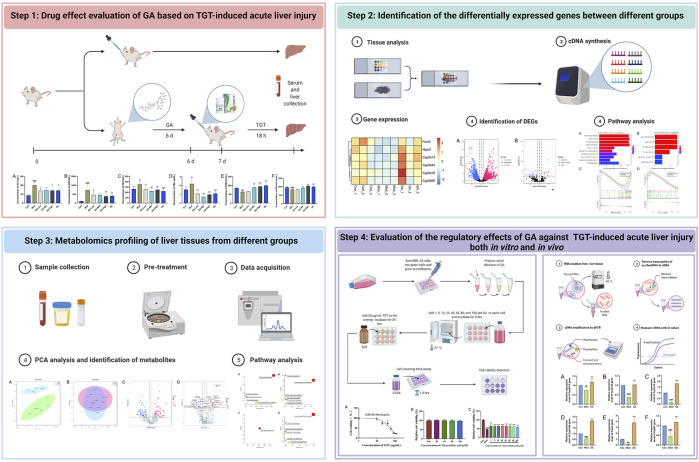


## Introduction

TGT, as the preparations of the main chemical constituents derived from *Tripterygium wilfordii* Hook. f. (TW), has been recognized by WHO as “China pioneered novel plant drugs” and was regarded as the most commonly used Chinese patent drug in rheumatoid arthritis (RA) treatment (Jiang et al., 2021). However, liver damage within a narrow dosage window (Lin et al., 2020) limits its further broad and safe application (Luo et al., 2017). Data from the National Center for Adverse Reaction Monitoring of China showed that from September 2014 to September 2019, a total of 472 cases of adverse reactions of TGT were reported, including 54 cases of abnormal liver function, accounting for 11.44% (Lin et al., 2020). Clinical reports also indicate that TGT-induced liver damage is mainly caused by liver parenchymal cell damage, similar to acute viral liver injury (Luo et al., 2017). Thus, it is urgent to discover drugs to improve its safe use in clinical settings.

The main treatment strategy for TGT-induced liver injury is to discontinue or reduce TGT and avoid re-exposure. In the practice of TCM, herb synergy is an important means to reduce toxicity and increase the efficacy of herbs. Previous research discovered that the roots of *Glycyrrhiza glabra* L. (licorice) could reduce the liver toxicity of TW and increase its therapeutic effect against RA (Ma et al., 2017).

GA, the major bioactive ingredient in licorice, shows hepatoprotective, antioxidant, anti-inflammatory, and immunoregulatory activities (Gong et al., 2014; Gu et al., 2014; Hou et al., 2014; Hsiang et al., 2015; Sil et al., 2015). The liver protection effect of GA was partly achieved by inhibiting hepatocyte apoptosis and activating hepatic stellate cells (Yan et al., 2016). Thus, GA can be regarded as a potential drug to improve the safe application of TGT, but the characteristics of its pharmacodynamics and mechanisms still need further investigation.

This study made effort to probe into the protective effect of GA in alleviating TGT-induced acute liver injury and to clarify its possible mechanisms through transcriptome and metabolome association analyses.

## Materials and Methods

### TGT-Induced Acute Liver Injury Model and Treatment

This study was approved by the Institutional Animal Care and Use Committee and Animal Ethics Committee of the Institute of Chinese Materia Medica, with approval number 2021B110, and the *in vivo* experiments were performed in accordance with the National Institutes of Health (NIH) Guidelines (NIH publication 86-23, revised 1985). Sixty male (KM) mice were bought from Beijing Vital River Laboratory Animal Technology Co. Ltd. (Beijing, China) with Animal Qualification Certificate No. SYXK (Beijing) 2019-0003 and adapted to the surroundings for 3 days before they were randomly distributed into six groups (n = 10), namely, Con, Mod, GA-Low, Mid, High, and GC.

The TGT-induced acute liver injury was constructed; in short, TGT at a dosage of 270 mg/kg (20 times of the clinical daily dose) was orally administrated when the 6 day treatment was completed, and animals were sacrificed 18 h later. In this study, all drugs were pretreated for six successive days. Ganlixin capsule (GC) was regarded as a positive drug with a dosage of 75 mg/kg by oral administration, which is based on clinical application and literature reports (Gao et al., 2019); the low, middle, and high dosages of GA were 25, 50, and 100 mg/kg by intraperitoneal injection, which were selected according to previous studies (Wang et al., 2017); the control group was given the same volume of saline.

TGT was purchased from Zhejiang DND Pharmaceutical Co., Ltd. (product code number approved by SFDA: Z33020422); GC was purchased from Chia Tai Tianqing Pharmaceutical Group Co., Ltd. (product code number approved by SFDA: H10940191); GA was provided by Shanghai Standard Technology Co., Ltd. (product code: ST00660120).

The blood samples were obtained from the abdominal aorta and let stand for 1 h and then centrifuged at 3,500 rpm for 10 min to get the serum, which was then stored at −80°C for liver function index level analysis. Livers were collected for HE staining, lipid metabolomics, transcriptome analysis, and experimental validation.

### Serum Sample Assays

Serum contents of ALT, AST, ALP, TBIL, SOD, and GSH-Px were quantified by a model 120 automatic biochemical analyzer (Toshiba, Japan) from Bei Jian Xin Chuang yuan Biotechnology Co., Ltd (Beijing).

### Histology

Liver tissues were fixed in 4% paraformaldehyde for 1 day and then embedded in paraffin and cut into sections (5 μm) before being stained with HE to observe for pathological by microscope; photos in this paper were of ×40 magnification.

### Sample Preparation, Library Construction, and Clustering

Con, Mod, and GA (middle group) samples were used for transcriptome sequencing (n = 3), at Maiwei Metabolic Biotechnology Co., Ltd (Jiaxing). Samples were cleaned by a QIAquick PCR kit. The cDNA fragments were separated by agarose gel electrophoresis, and then fragments of 100 ± 300 bp were further enriched to create cDNA libraries. Clustering was performed using a TruSeq PE Cluster Kit v3-cBot-HS (Illumina). The library was sequenced using an Illumina HiSeq platform; then 125 bp/150 bp paired-end reads were generated.

### Transcriptome Data Analysis

Raw data of fastq format were processed by in-house Perl scripts; clean data (clean reads) were obtained after removing redundancy in Q20, Q30, and GC contents. The index of the reference genome was built using Bowtie v2.2.3, and paired-end clean reads were aligned to the reference genome using TopHat v2.0.12. Read number was counted by HTSeq v0.6.1; then fragments per kilobase of transcript per million fragments that mapped each gene were calculated. Differential expression analysis of different biological conditions was performed using the edgeR. The *p*-values were adjusted according to the Benjamini and Hochberg approach. Genes with an adjusted |log2Fold Change| ≥1 and a *p*-value <0.05 were regarded as differentially expressed, and the “cluster Profiler” software package 1 (version 3.14.3) and “org.Mm.eg.db” annotation file (version 3.10.0) of the R programming language were used for KEGG enrichment. The downregulated genes of “Mod vs. Con” and the upregulated genes of “GA vs. Mod” were analyzed, and the top 10 KEGG functional pathways were selected and visualized. Then the overlap result was taken for further gene set enrichment analysis (GSEA).

### RNA-Seq Data Validation

RNA-seq data were validated by quantitative PCR, which was performed on a real-time PCR system (LightCycler 96, Roche, Switzerland) using a ×2 RealStar Green Fast Mixture (Genstar, China). Added for each reaction was 1.25 μl of the forward and reverse primers as well as 1 μl of the cDNA template. The relative expression quantitative analysis was performed using the 2^−ΔΔCt^ method. The primer information we used in the qPCR analysis of this study was provided in Supplementary Table S1.

### Metabolome Data Analysis

Liver samples (n = 6, each group) were prepared and detected according to the metabolome analysis protocol of Maiwei Metabolic Biotechnology Co., Ltd (Jiaxing). Unsupervised principal component analysis (PCA) as well as orthogonal partial least squares discrimination analysis (OPLS-DA) was performed by the statistics function pr comp within R. Unsupervised PCA was carried out after scaling the unit variance. The data were log-transformed (logϵ) and mean-centered before OPLS-DA. VIP values were extracted from the OPLS-DA result, which also includes score plots and permutation plots generated by the R package MetaboAnalystR. Metabolites with a VIP ≥1 and an absolute LogϵFC (fold change) ≥1 were regarded as significantly regulated metabolites. Identified metabolites were annotated, mapped to the KEGG pathway database and further imported into the metabolite set enrichment analysis.

### Cell Culture and Treatment

Rat hepatocytes (BRL-3A) were purchased from China Infrastructure of Cell Line Resources. They were maintained by DMEM, high glucose, and 10% FBS according to the manufacturer’s recommendation at 37°C in a humidified atmosphere with 5% CO_2_. When BRL-3A reached 80%–90% confluence, they were exposed to TGT, and their IC50 values were calculated so as to select the toxicity model construction dose. The liver protective effect evaluation of GA also set different groups: control and GA groups (with 1, 5, 10, 20, 40, 60, 80, and 100 μM, respectively). The control group was given the same operation without drugs. Next, its concentration range was narrowed within the safe and effective range. The cell viability of BRL-3A was evaluated by a Cell Counting Kit-8 assay (KeyGen Biotech Co., Ltd. Nanjing) based on the manufacturer’s instructions.

### Statistical Analysis

Data analysis was performed by SPSS software (version 19.0, SPSS Institute Inc., Chicago, IL). One-way repeated-measures analysis of variance (ANOVA) was used to analyze differences between groups. Statistics was presented as mean ± standard deviation (SD) by GraphPad Prism software (version 8.0, San Diego, CA, United States). Differences with a *p*-value less than 0.05 were regarded as statistically significant.

## Results

### GA Effectively Alleviates TGT-Induced Acute Liver Injury in Mice

**FIGURE 1:**
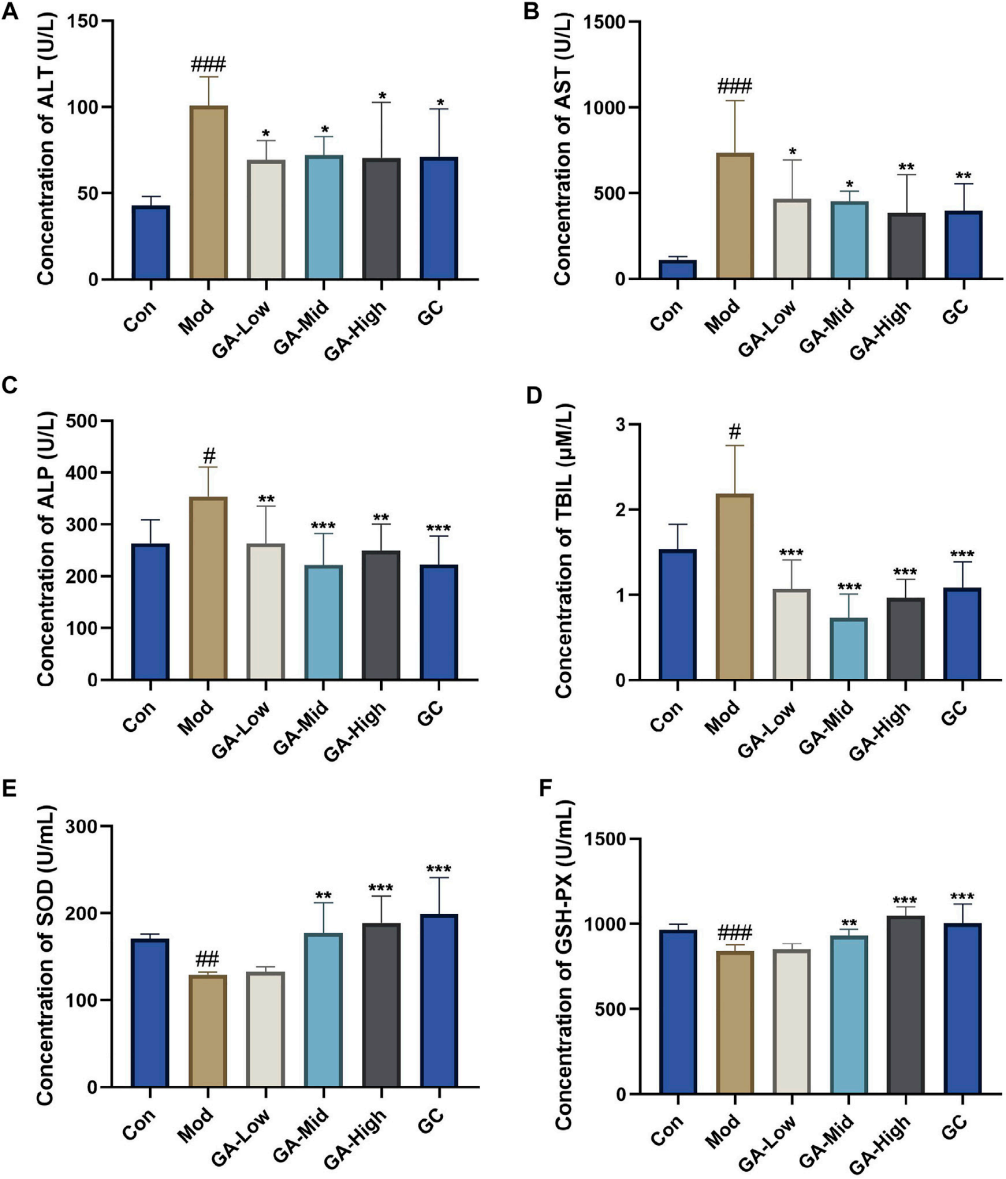
Influence of GA on the serum biochemical parameters in TGT-induced acute liver injury mice. (**A–F**) Serum concentrations of ALT, AST, ALP, TBIL, SOD, and GSH-PX in mice of different groups. Con, normal control; Mod, model; GA-Low, glycyrrhizic acid low dose; GA-Mid, glycyrrhizic acid medium dose; GA-High, glycyrrhizic acid high dose; GC, positive drug diammonium glycyrrhizinate capsules group (same below). **p < 0.01, ***p < 0.001 “GA vs. Mod”; ^#^ *p* < 0.05, ^##^ *p* < 0.01, ^###^ *p* < 0.05 “Mod vs. Con.”

As shown in Figure 1, in contrast to that in the control group, serum contents of ALT, AST, ALP, and TBIL of mice in the TGT model group were significantly increased, whereas GA reduced them to varying degrees; data had statistical differences in the GA-mid dosage. The serum concentrations of SOD and GSH-PX in TGT model mice were abnormally decreased, and GA at the middle dosage significantly increased them.

### GA Significantly Improved the Liver Morphology of TGT-Induced Acute Liver Injury Mice

**FIGURE 2:**
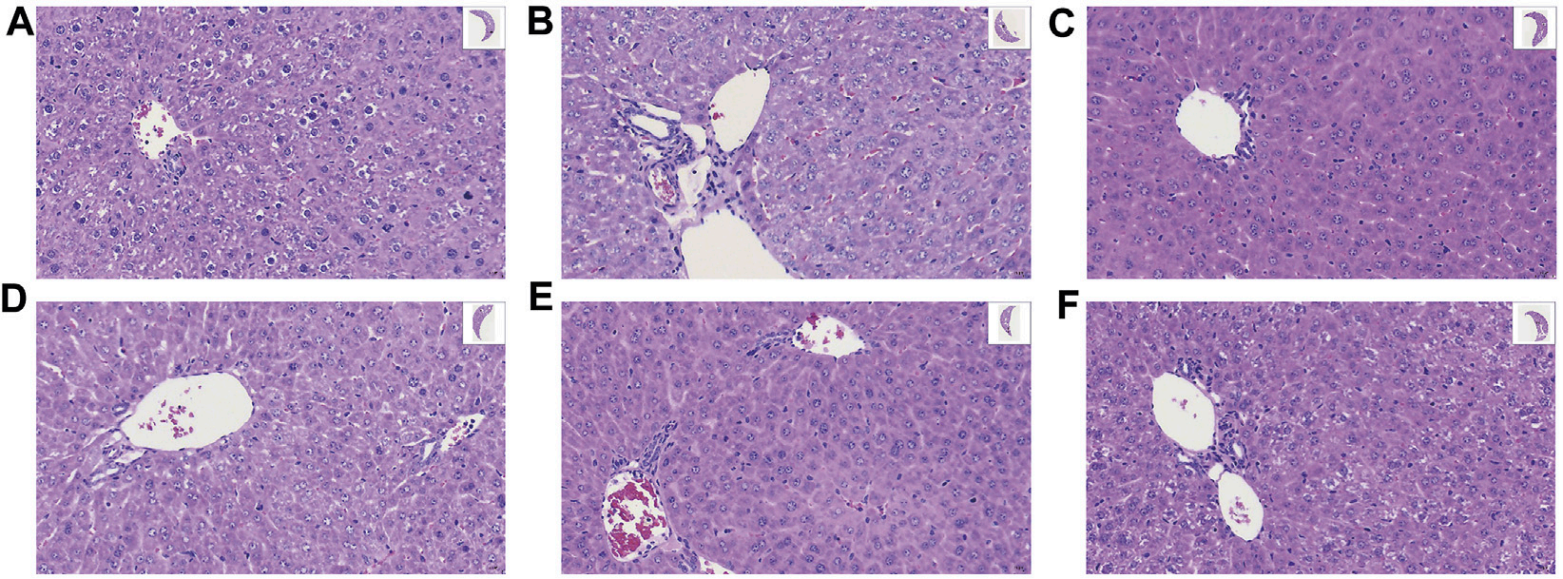
Effect of GA on liver histopathology of TGT-induced acute liver injury (×40). (**A–F**) Con, Mod, GC, GA-Low/Mid/High groups.

HE staining (Figure 2) indicated that the normal mice had intact liver tissue with clear hepatic lobule and regular arrangement of hepatocyte; in the Mod group, the basic structure of hepatocytes was found to be lost, showing significant steatosis, inflammatory cell infiltration, and swelling of hepatocytes; in contrast, GA significantly improved the liver morphology; in the GA-mid and high groups, especially, only a slight steatosis was found. These results suggested that GA attenuated the detrimental effects of TGT on the liver.

### GA Markedly Regulates CYP2 and CYP3A in the Liver of Mice with TGT-Induced Acute Liver Injury

**FIGURE 3:**
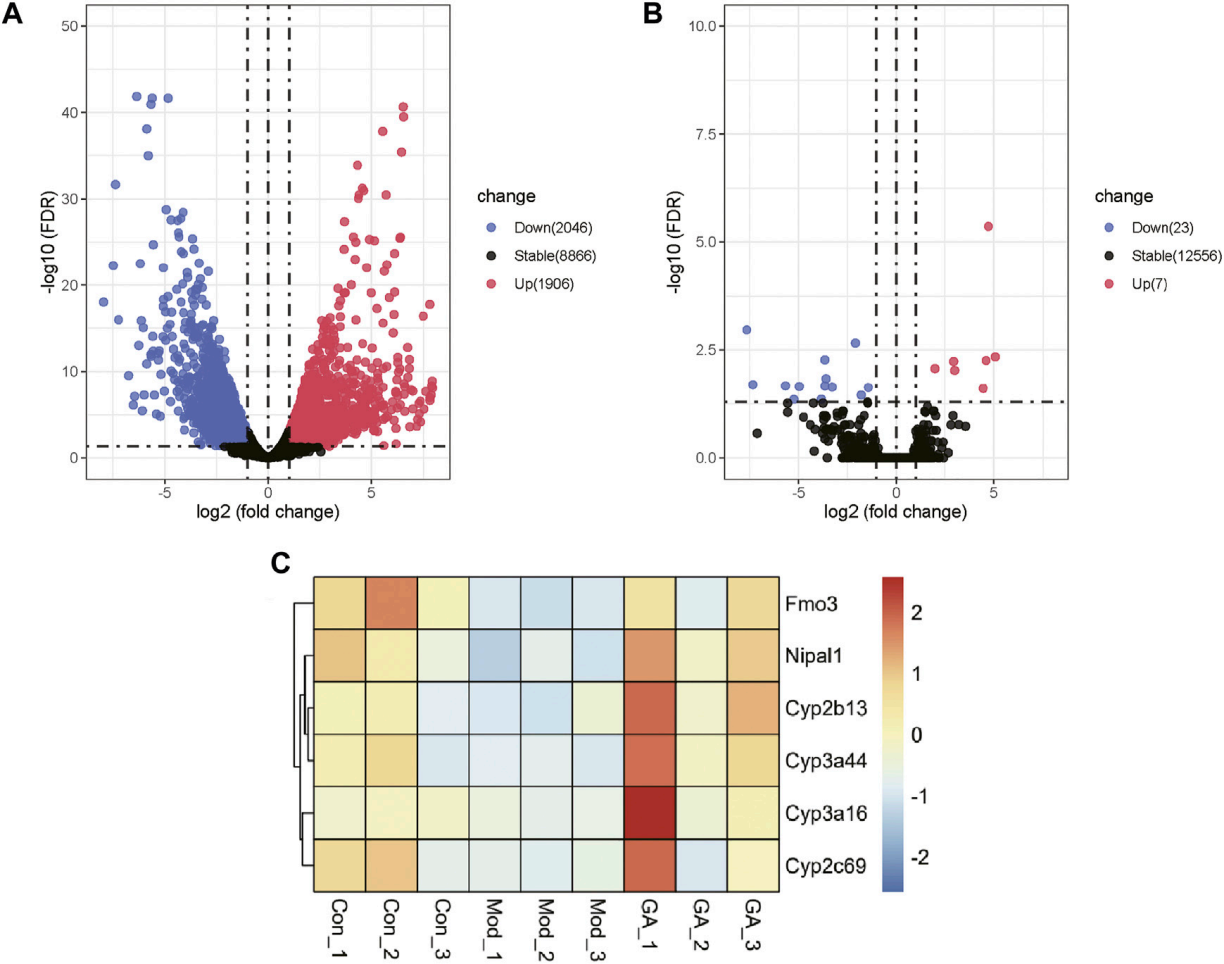
Expression profiling changes of genes in the liver of Con, Mod, and GA groups. Volcano plot indicating upregulated and downregulated genes. (**A**) Volcano map showing the DEGs of “Mod vs. Con”; (**B**) volcano map showing the DEGs of “GA vs. Mod”; (**C**) heat map showing overlap genes of the downregulated genes in the “Mod vs. Con” and the upregulated gene set in the “GA vs. Mod.”

The differentially expressed genes (DEGs) between the “Con vs. Mod” and “Mod vs. GA” obtained by transcriptomic data are shown in the volcano and heatmap plots (Figure 3). Compared with the Con group, there are 1,906 upregulated genes and 2,046 downregulated genes in the Mod group (Figure 3A). Besides, 7 upregulated and 23 downregulated DEGs were obtained in “GA vs. Mod” (Figure 3B). Then we intersected the downregulated genes in the “Mod vs. Con” with the upregulated gene set in the “GA vs. Mod,” and six differential genes were obtained, namely, Cyp2b13, Cyp2c69, Cyp3a16, Cyp3a44, Fmo3, and Nipal1 (Figure 3C). Thereinto, Cyp2b13 and Cyp2c69 belong to CYP2, whereas Cyp3a44 and Cyp3a16 are members of CYP3A, indicating that GA markedly regulates CYP2 and CYP3A in the liver of mice with TGT-induced acute liver injury.

**FIGURE 4:**
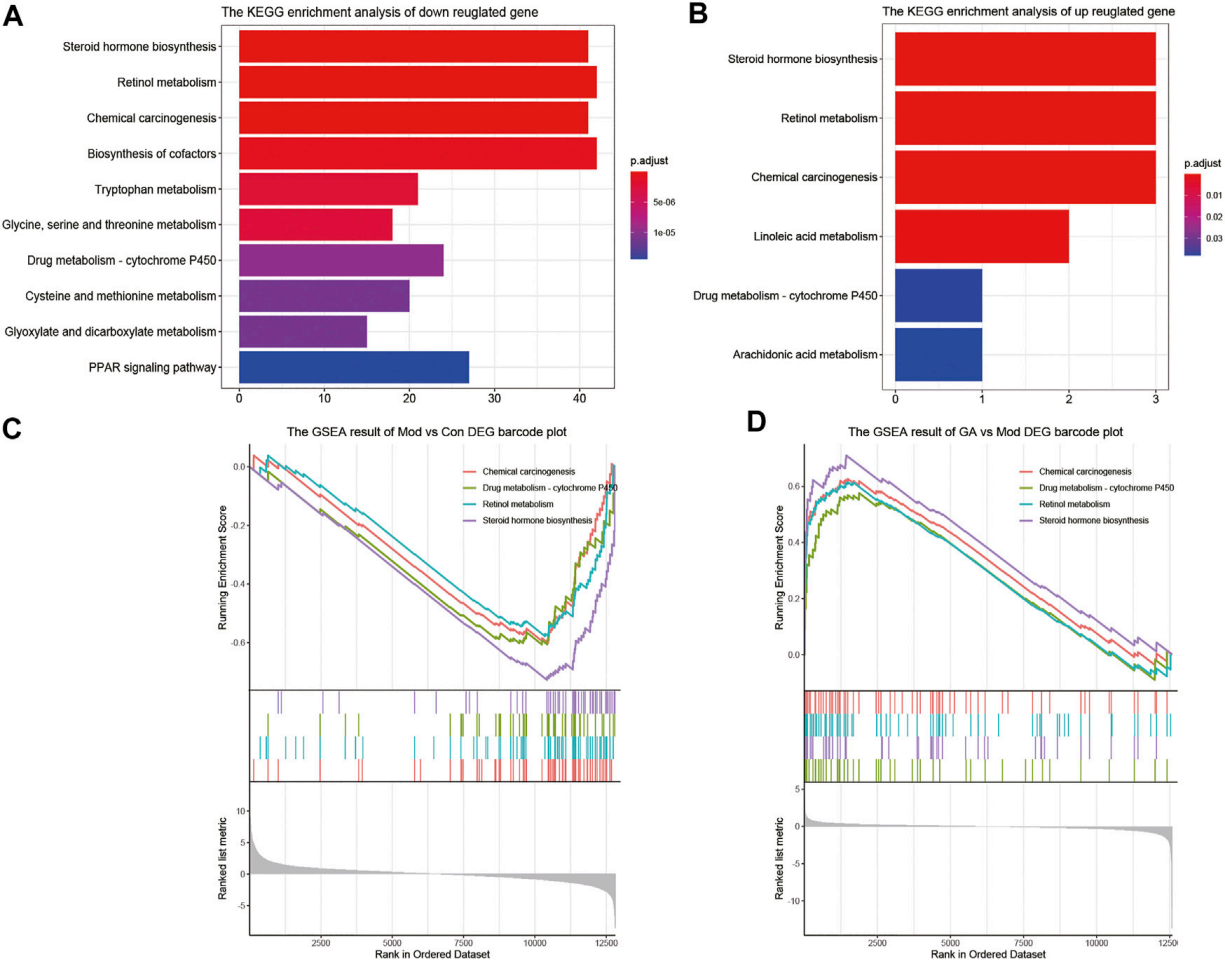
Significantly enriched pathways between “Mod vs. Con” and “GA vs. Mod.” (**A**) The KEGG enrichment result of downregulated genes in “Mod vs. Con”; **(B)** the KEGG enrichment result of upregulated genes in “GA vs. Mod”; (**C**) the GSEA enrichment result of “Mod vs. Con”; (**D**) the GSEA enrichment result of “GA vs. Mod.”

As shown in Figures 4A,B, “steroid hormone biosynthesis,” “retinol metabolism,” “chemical carcinogenesis,” and “drug metabolism-cytochrome P450” were the top four signaling pathways with high enrichment scores. Moreover, the GSEA enrichment results (Figures 4C,D) also indicate that the above four pathways were markedly downregulated in the “Mod vs. Con” (*p* < 0.05) and significantly upregulated in the “GA vs. Mod” (*p* < 0.05). Thus, GA exerted a liver protective effect *via* a multi-target and multi-pathway manner.

### qRT-PCR Validation of Key Transcriptomic Data

**FIGURE 5:**
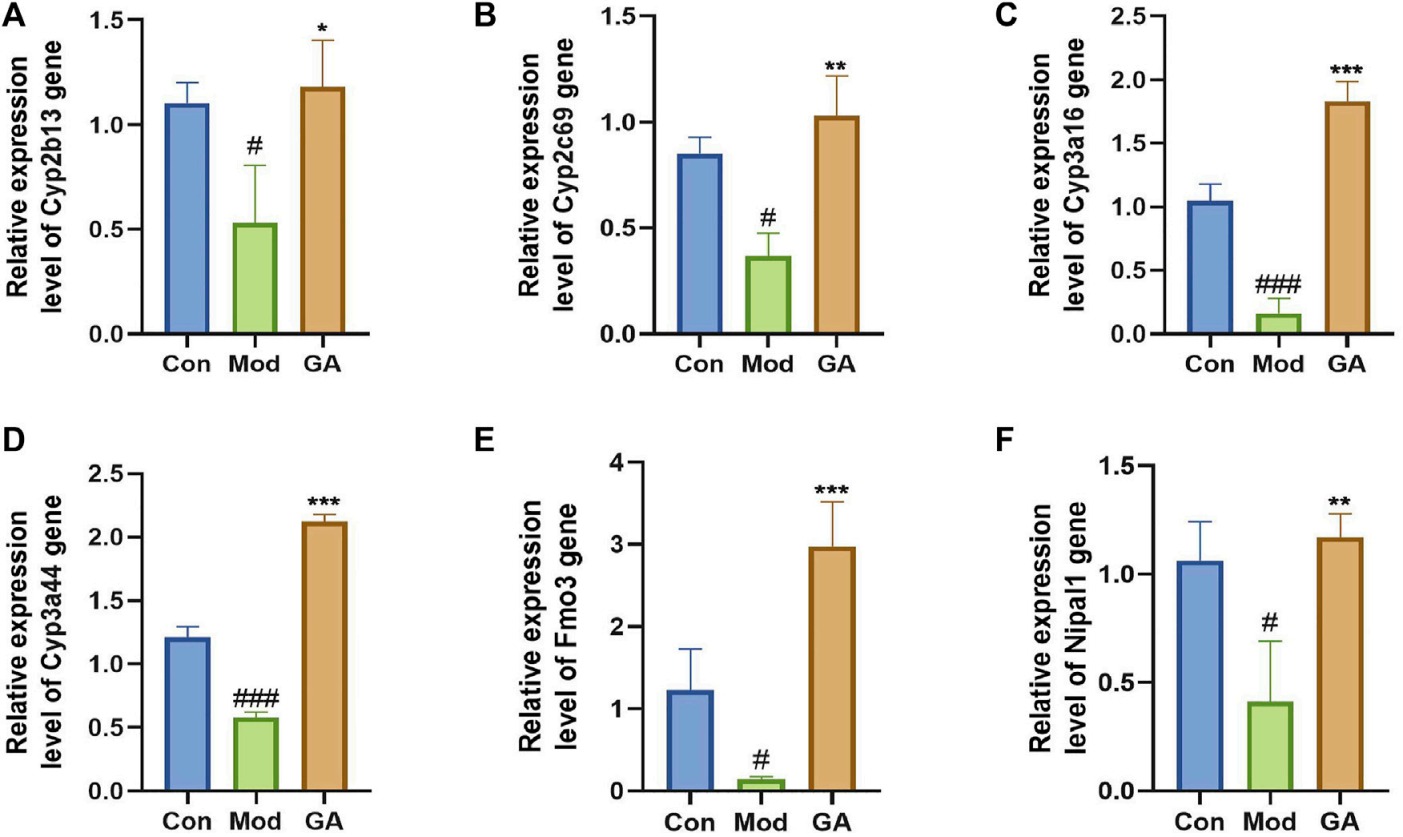
qRT-PCR validation of significant DEGs. (**A–F**) Relative expression level of Cyp2b13, Cyp2c69, Cyp3a16, Cyp3a44, Fmo3, and Nipal1 genes. The qRT-PCR data were presented as the mean ± SD (n = 3); *p < 0.05, **p < 0.01, ***p < 0.001 “GA vs. Mod”; ^#^ *p* < 0.05, ^###^ *p* < 0.05 “Mod vs. Con.”

The expression levels of Cyp2b13, Cyp2c69, Cyp3a16, Cyp3a44, Fmo3, and Nipal1 were detected to verify the accuracy of RNA-seq results (Figure 5). After intervention with TGT, the gene levels of Cyp2b13, Cyp2c69, Cyp3a16, Cyp3a44, Fmo3, and Nipal1 were significantly downregulated by 51.69%, 56.58%, 84.56%, 52.02%, 88.05%, and 61.20%. In contrast to the model group, GA significantly upregulated the gene levels by 121.64%, 179.60%, 1030.41%, 265.37%, 1921.74%, and 183.43%. All validation results were in accordance with the transcriptome sequencing data.

### Differentially Accumulated Metabolites of Samples

**FIGURE 6:**
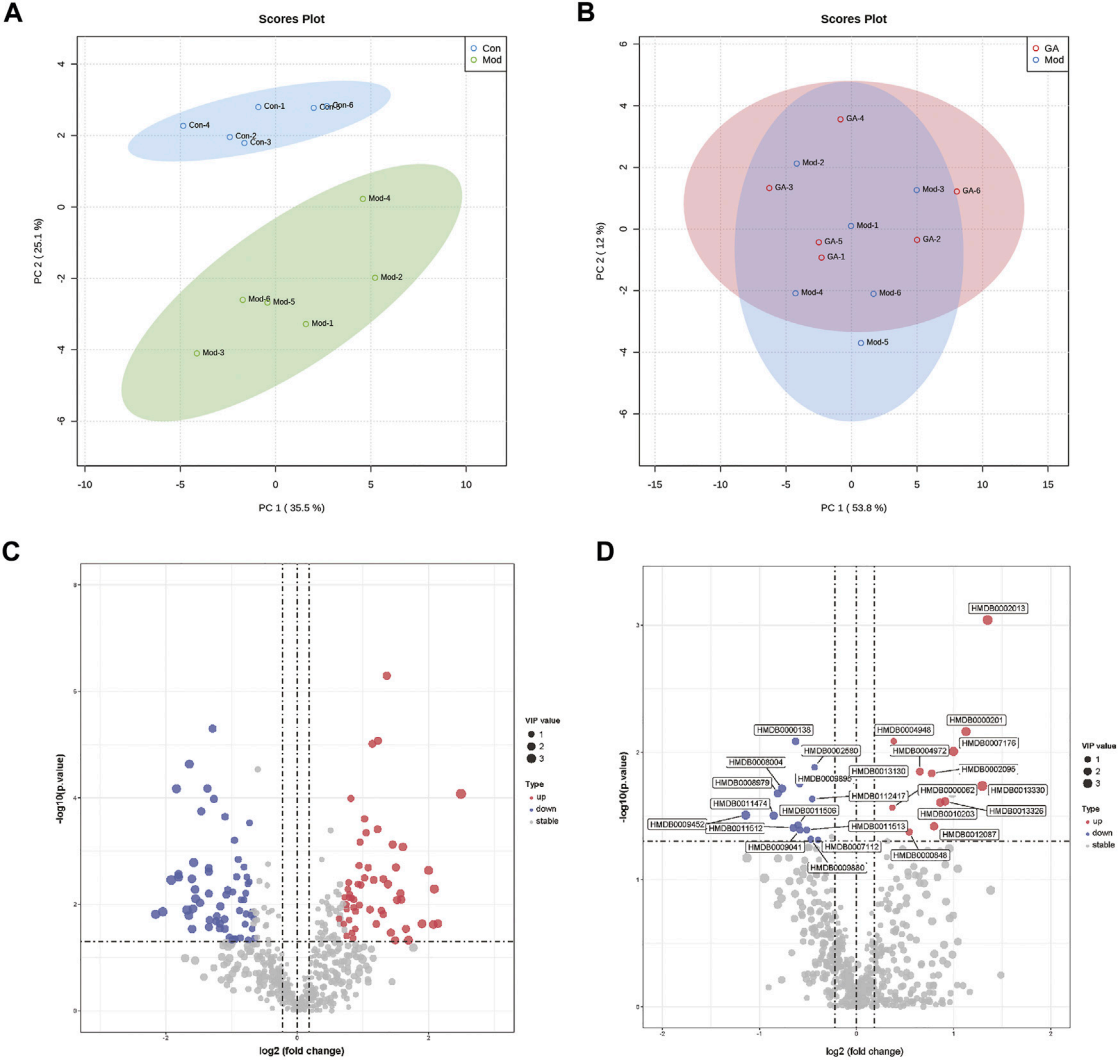
Metabolomics profiling of liver tissues from different groups. (**A**) PCA plots of “Mod vs. Con”; (**B**) PCA plots of “GA vs. Mod”; (**C**) volcano map of “Mod vs. Con”; **(D)** volcano map of “GA vs. Mod.”

As shown in Figures 6A,B, the metabolites of “Mod vs. Con” and “GA vs. Mod” were clustered based on PCA, respectively. Significant differences existed in the metabolism of Mod and Con groups, and there were slight differences in metabolites within GA and Mod groups. The OPLS-DA score plot showed a significant separation effect between samples of Mod and Con groups (Supplementary Figure S1).

Differentially accumulated metabolites were screened based on the following standards: upregulated, VIP ≥ 1, *p* ≤ 0.05, fold change ≥ 1.2; and downregulated, VIP ≥ 1, *p* ≤ 0.05, fold change ≤ 0.8. As a result, 116 (“Mod vs. Con”) metabolites were obtained; among them, 55 were upregulated (red) and 61 were downregulated (blue) (Supplementary Table S2); 29 metabolites (“GA vs. Mod”) were identified, of which the upregulated and downregulated numbers were 15 and 14, respectively (Figures 6C,D).

**FIGURE 7:**
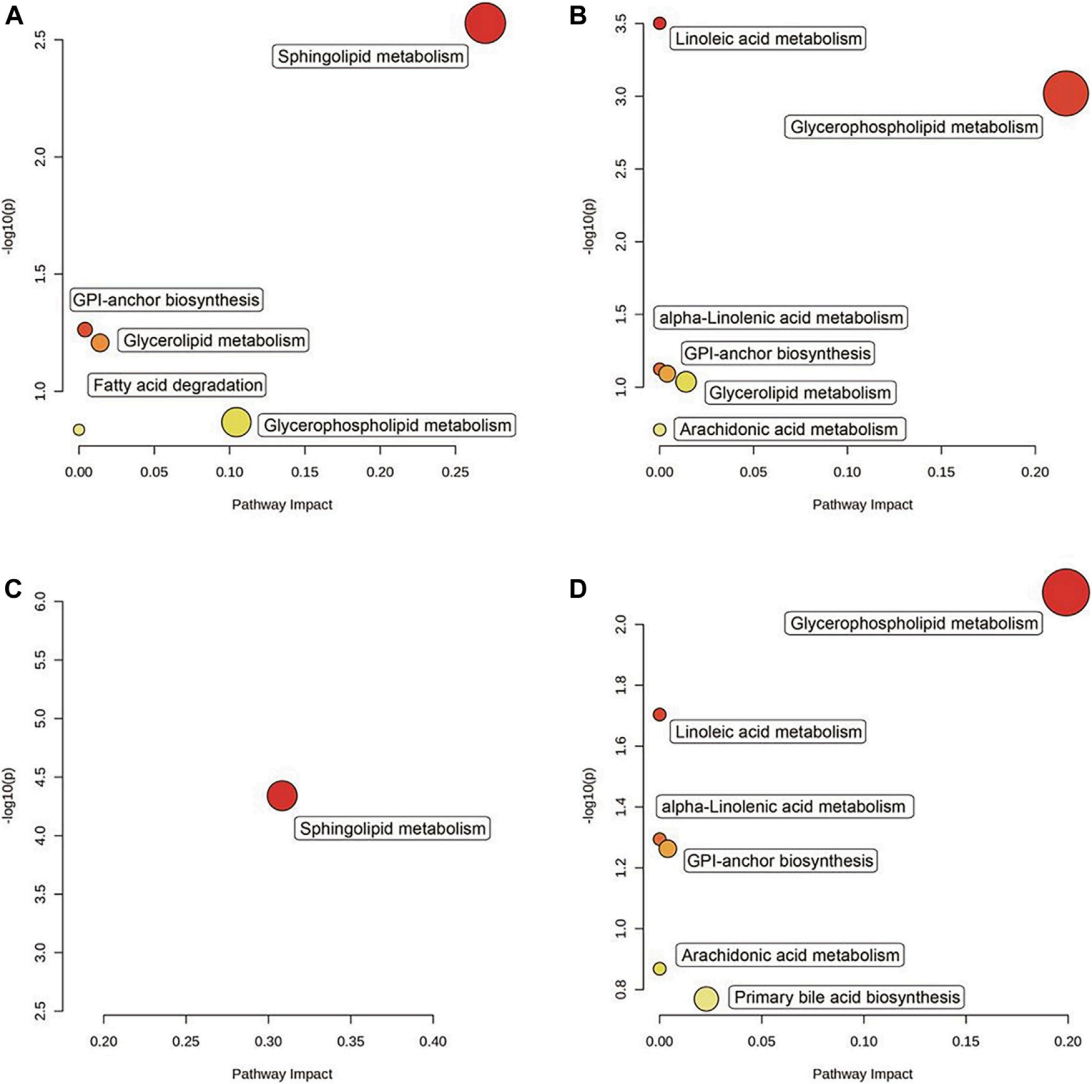
Significantly enriched pathways between “Mod vs. Con” and “GA vs. Mod.” The KEGG enrichment resulting from the screening of (**A**) upregulated genes in the “Mod vs. Con,” (**B**) downregulated genes in the “Mod vs. Con,” (**C**) upregulated genes in the “GA vs. Mod,” and (**D**) downregulated genes in the “GA vs. Mod.”

Based on the KEGG analysis result, five pathways were upregulated (Figure 7A), and six pathways were downregulated (Figure 7B) in the “Mod vs. Con,” whereas one pathway was upregulated (Figure 7C) and six pathways were downregulated (Figure 7D) in the “GA vs. Mod.” Among them, the “glycerophospholipid metabolism” pathway and the “glycosylphosphatidylinositol (GPI)–anchor biosynthesis” pathway were upregulated in the “Mod vs. Con” and downregulated in the “GA vs. Mod.”

### GA Pre-administration Effectively Reduces TGT-Induced Hepatocyte Toxicity *In Vitro*

**FIGURE 8:**
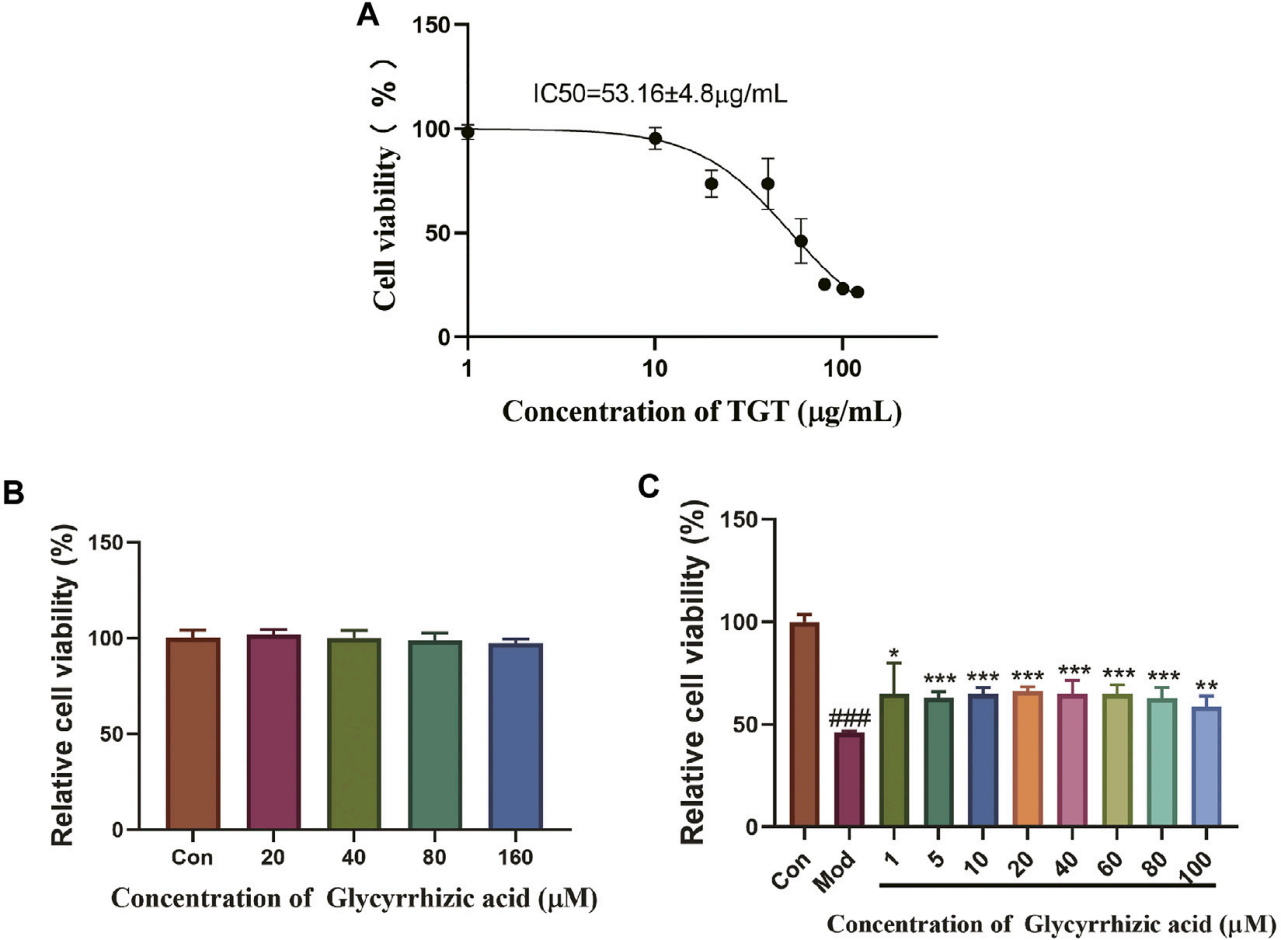
Protective effect of GA acting on TGT-induced acute liver injury *in vitro*. (**A**) Influence of TGT on BRL-3A cell viability; (**B**) safe dose screening of GA; (**C**) effect of GA on TGT-induced BRL-3A hepatocyte toxicity.

Based on BRL-3A, the hepatocytotoxicity of TGT was detected; as a result, the survival rate of hepatocytes decreased to 50% at a working concentration of about 50 μg/ml of TGT (Figure 8A); thus, we use TGT at this dosage to construct an acute liver injury model *in vitro*. In addition, the individual use of GA showed no significant hepatocyte toxicity on BRL-3A under the concentration of 160 μM (Figure 8B). Therefore, concentrations of 1, 5, 10, 20, 40, 60, 80, and 100 μM were selected for subsequent tests. The results showed that pre-administration of GA for 6 h, followed by TGT induction for 24 h, markedly alleviated the inhibitory effect of TGT on BRL-3A activity (Figure 8C).

## Discussion

TGT was a commercial drug derived from TW mainly used in the clinical treatment of RA (Yang et al., 2019). However, the severe toxicity and side effects, such as hepatotoxicity, have become an obstacle to its broad application (Tian et al., 2019). Licorice, a classical traditional Chinese herb, can be effectively used in combination with drugs including TW to reduce toxicity and increase efficacy (Kao et al., 2013; Richard, 2021). According to the International Serious Adverse Reactions Association, serum contents of ALT and ALP or the combination of ALT and TBIL levels can be regarded as biomarkers in the evaluation of the existence and progression of drug-induced liver injury (Xia and Fu, 2017). GA, the major active constituent of licorice, has been reported to significantly decrease and normalize the enzyme activities of AST and ALT in serum of patients with hepatitis (Li et al., 2015).

In this study, we validated the liver protection effect of GA based on TGT-induced acute liver injury models both *in vivo* and *in vitro*. GA could effectively alleviate TGT-induced acute liver injury in mice by reducing the concentrations of ALT, AST, ALP, and TBIL, increasing the contents of oxidative stress-related indicators SOD and GSH-Px, and reducing the severity of the liver tissue pathology change. *In vitro* data indicate that the inhibition effect of TGT for 24 h on BRL-3A cell viability could be alleviated by GA pre-administration for 6 h.

Based on transcriptome results, we identified six DEGs, namely, Cyp2b13, Cyp2c69, Cyp3a16, Cyp3a44, Fmo3, and Nipal1, most of which were enriched in drug metabolism–cytochrome P450 pathways. All of these genes, except Nipal1, are involved in drug metabolism in the liver. Fmo3 is an important liver microsomal enzyme involved in the oxidative metabolism of drugs, exogenous substances, and other chemicals *in vivo*. CYP3A4, as the most important metabolizing enzyme, metabolizes the largest number of drugs among the CYP450 family members, accounting for about 50% of the total number of drugs metabolized by CYP450, and is involved in multiple drug metabolic interactions; the CYP2 family metabolizes drugs second only to the CYP3A family (Liao and Chen, 2012). Interestingly, GA plays a detoxification role in changing the characteristics of drug metabolism through the regulation of CYP450 family members, which is related to the compatible drugs (Xu et al., 2017). To sum up, our study indicates that GA may ameliorate TGT-induced acute liver injury by regulating drug metabolism in the liver.

In addition, metabonomics results indicate that the differential metabolites of “Mod vs. Con” and “GA vs. Mod” were significantly enriched in GPI-anchor biosynthesis and glycerophospholipid metabolism pathways. GPI-anchoring modification is one of the common post-translational modification methods of eukaryotic cell membrane surface proteins. Glycoprotein and glycolipid synthesis is one of the main functions of the liver. GPI anchors the protein outside of the cell plasma membrane through glycosylation. “GPI-anchor biosynthesis” disorder leads to abnormal glycosylation, which affects the structure and function of the liver (Abuduxikuer and Wang, 2019). Glycerophospholipid, as one of the constituents of the cell membrane, participates in the recognition and signal transduction of the cell membrane to the protein and affects drug phase I metabolic enzyme cytochrome P450s and phase II metabolic enzyme UDP-glucuronosyltransferase, aggravating metabolite disorder and leading to liver disease (Stingl et al., 2014). The differential metabolites were classified as phosphatidylcholine (PC) and phosphatidylethanolamine (PE). In the liver, PC is the most important ingredient of the cellular membrane, whereas PE regulates membrane fusion and provides ethanolamine for glycosylphosphatidylinositol anchors of cell surface proteins. They are important in phospholipid metabolism and signal transduction, which contributes to the development of drug-induced liver injury, but the exact mechanism of injury has not been elucidated (Ming et al., 2017). Researchers have observed the reconstruction of PC/PE in rat liver with TW-caused liver injury (Xie et al., 2016). We also discovered a variety of PC and PE species in TGT-injured livers, indicating that energy lipid modification, membrane reconstruction, and potential signaling lipid alterations were closely involved in the development and treatment of liver injury.

## Conclusion

In conclusion, this study preliminarily investigated the pharmacodynamic characteristics of GA in alleviating TGT-induced acute liver injury. We clarified the upregulation of GA on CYP3A and CYP2 expression levels; also, the metabolic conversion of TGT induced metabolic abnormalities in PC and PE, thus suggesting the potential of GA as a promising candidate for improving the clinical safety of TGT. Further investigations are required to reveal the underlying regulation mechanisms of GA on TGT-induced acute liver injury in molecular and compound levels.

## Data Availability

The original contributions presented in the study are included in the article/Supplementary Material, further inquiries can be directed to the corresponding authors.

## Abbreviations

ALP, alkaline phosphatase; ALT, alanine aminotransferase; AST, aspartate aminotransferase; BRL-3A, rat hepatocyte; DEGs, differentially expressed genes; GA, glycyrrhizic acid; GPI, glycosylphosphatidylinositol; GSEA, gene set enrichment analysis; GSH-PX, glutathione peroxidase; HCA, hierarchical cluster analysis; HE, hematoxylin–eosin; KEGG, Kyoto Encyclopedia of Genes and Genomes; OPLS-DA, orthogonal partial least squares discrimination analysis; PC, phosphatidylcholine; PCA, principal component analysis; PCC, Pearson correlation coefficients; PE, phosphatidylethanolamine; PPI, protein–protein interaction; RA, rheumatoid arthritis; SOD, superoxide dismutase; TBIL, total bilirubin; TCM, traditional Chinese medicine; TG, tripterygium glycosides; TGT, tripterygium glycosides tablet; TW, *Tripterygium wilfordii* Hook. f.
