# Supplementary material for: Transcriptome and Lipid Metabolomics-Based Discovery: Glycyrrhizic Acid Alleviates Tripterygium Glycoside Tablet-Induced Acute Liver Injury by Regulating the Activities of CYP and the Metabolism of Phosphoglycerides
Source: Front Pharmacol. 2022 Feb 14;12:822154. doi: 10.3389/fphar.2021.822154 (PMC8883433; doi:10.3389/fphar.2021.822154)
Supplement: Supplementary file 1 [file DataSheet1.docx]

Supplementary Material

## Supplementary Tables

# Table S1 Sequences of primers for RT-PCR

| Gene | Sequences (5’-3’) | Source |
| --- | --- | --- |
| GAPDH F | CGTGCCGCCTGGAGAAACC | This manuscript |
| GAPDH R | TGGAAGAGTGGGAGTTGCTGTTG |  |
| Mu-FMO3 F | GGAAGAGTTGGTGAAGACCG | Rudraiah et al.  (2014) |
| Mu-FMO3 R | CCCACATGCTTTGAGAGGAG |  |
| Mu-Nipal1 F | GTCAGAGTCGCTGCCTTATCC | PrimerBank |
| Mu-Nipal1 R | TGCAAGAGACCCTTCTTTTTGAG |  |
| Mu-Cyp2b13 F | TTTTCTTCCAGTGTGTTACAGCC | PrimerBank |
| Mu-Cyp2b13 R | AACGCAGGAACTGTTCATCTG |  |
| Mu-Cyp2c69 F | TGTAGTCTTGGTGCTTTGTCTG | PrimerBank |
| Mu-Cyp2c69 R | ACAATAGGCTGTGAGCCAAAATA |  |
| Mu-Cyp3a16 F | TTCAGCGCTCTCACTGGATA | Graeser et al.  (2011) |
| Mu-Cyp3a16 R | ACCCCCACGTTTTTCCATAC |  |
| Mu-Cyp3a44 F | TTGTGGAGGAAGCCAAAAAGTTT | Wiwi et al.  (2004) |
| Mu-Cyp3a44 R | TGAGAAGAGCAAAGGATCAAAAAAGT |  |

F: forward; R: reverse

# Table S2 Identification results of differential metabolites of “Mod vs. Con”

| **HMDB** | **Compounds** | **Class.I** | **type** |
| --- | --- | --- | --- |
| **HMDB0000062** | DL-Carnitine | FA | up |
| **HMDB0000222** | Carnitine C16:0 | FA | up |
| **HMDB0000651** | Carnitine C10:0 | FA | up |
| **HMDB0000705** | Carnitine C6:0 | FA | up |
| **HMDB0001348** | SM(d18:1/18:0) | SL | up |
| **HMDB0004950** | Cer(d18:1/18:0) | SL | up |
| **HMDB0005462** | TG(18:1_18:2_20:4) | GL | up |
| **HMDB0005476** | TG(18:2_20:4_20:4) | GL | up |
| **HMDB0006469** | Carnitine C18:2 | FA | up |
| **HMDB0008923** | PE(16:0_16:0) | GP | up |
| **HMDB0008925** | PE(16:0_18:0) | GP | up |
| **HMDB0008927** | PE(16:0_18:1) | GP | up |
| **HMDB0008958** | PE(16:1_18:0) | GP | up |
| **HMDB0008991** | PE(18:0_18:0) | GP | up |
| **HMDB0008994** | PE(18:0_18:2) | GP | up |
| **HMDB0009057** | PE(18:1_18:0) | GP | up |
| **HMDB0009060** | PE(18:1_18:2) | GP | up |
| **HMDB0009124** | PE(18:3_18:1) | GP | up |
| **HMDB0009222** | PE(20:0_18:0) | GP | up |
| **HMDB0009253** | PE(20:1_16:0) | GP | up |
| **HMDB0009256** | PE(20:1_18:1) | GP | up |
| **HMDB0009286** | PE(20:2_16:0) | GP | up |
| **HMDB0009489** | PE(20:1_20:1) | GP | up |
| **HMDB0009489** | PE(18:1_22:1) | GP | up |
| **HMDB0010494** | TG(18:2_18:2_22:4) | GL | up |
| **HMDB0011476** | LPE(0:0/18:1) | GP | up |
| **HMDB0011483** | LPE(0:0/20:2) | GP | up |
| **HMDB0011760** | Cer(d18:0/16:0) | SL | up |
| **HMDB0011761** | Cer(d18:0/18:0) | SL | up |
| **HMDB0011764** | Cer(d18:0/20:0) | SL | up |
| **HMDB0011765** | Cer(d18:0/22:0) | SL | up |
| **HMDB0011769** | Cer(d18:0/24:1) | SL | up |
| **HMDB0012085** | SM(d18:0/14:0) | SL | up |
| **HMDB0012087** | SM(d18:0/18:0) | SL | up |
| **HMDB0012090** | SM(d18:0/20:0) | SL | up |
| **HMDB0012097** | SM(d18:1/14:0) | SL | up |
| **HMDB0012101** | SM(d18:1/18:1) | SL | up |
| **HMDB0013334** | Carnitine C16:2 | FA | up |
| **HMDB0013336** | Carnitine C16-OH | FA | up |
| **HMDB0013339** | Carnitine C18:1-OH | FA | up |
| **HMDB0044387** | TG(16:0_20:4_22:6) | GL | up |
| **HMDB0044639** | TG(16:0_22:6_22:6) | GL | up |
| **HMDB0044930** | TG(18:0_18:1_22:5) | GL | up |
| **HMDB0044936** | TG(18:0_18:1_22:6) | GL | up |
| **HMDB0044990** | TG(18:0_20:1_22:6) | GL | up |
| **HMDB0045206** | TG(18:0_20:4_22:6) | GL | up |
| **HMDB0047929** | TG(14:1_16:1_22:6) | GL | up |
| **HMDB0049336** | TG(18:1_20:3_22:4) | GL | up |
| **HMDB0049343** | TG(18:1_20:3_22:6) | GL | up |
| **HMDB0049399** | TG(18:1_18:2_22:4) | GL | up |
| **HMDB0049490** | TG(18:1_20:4_22:6) | GL | up |
| **HMDB0050602** | TG(20:1_18:2_22:6) | GL | up |
| **HMDB0052580** | TG(18:2_20:3_20:4) | GL | up |
| **HMDB0052739** | TG(18:2_22:5_22:6) | GL | up |
| **HMDB0112412** | PS(18:1_20:1) | GP | up |
| **HMDB0240610** | SM(d18:1/20:1) | SL | up |
| **HMDB0004385** | PGE2 | FA | down |
| **HMDB0004702** | 12,13-EpOME | FA | down |
| **HMDB0004702** | 9,10-EpOME | FA | down |
| **HMDB0005375** | TG(16:0_16:1_20:0) | GL | down |
| **HMDB0005378** | TG(16:0_16:1_20:1) | GL | down |
| **HMDB0005379** | TG(16:0_16:1_18:2) | GL | down |
| **HMDB0005382** | TG(16:0_18:1_18:1) | GL | down |
| **HMDB0005384** | TG(16:0_18:1_18:2) | GL | down |
| **HMDB0005390** | TG(16:0_18:2_18:2) | GL | down |
| **HMDB0005409** | TG(18:0_18:2_20:0) | GL | down |
| **HMDB0007043** | DG(14:0_18:2) | GL | down |
| **HMDB0007099** | DG(16:0_16:1) | GL | down |
| **HMDB0007102** | DG(16:0_18:1) | GL | down |
| **HMDB0007103** | DG(16:0_18:2) | GL | down |
| **HMDB0007105** | DG(16:0_18:3) | GL | down |
| **HMDB0007111** | DG(16:0_20:3) | GL | down |
| **HMDB0007141** | DG(16:1_20:4) | GL | down |
| **HMDB0007168** | DG(18:0_20:3) | GL | down |
| **HMDB0007170** | DG(18:0_20:4) | GL | down |
| **HMDB0007219** | DG(18:1_18:2) | GL | down |
| **HMDB0007876** | PC(14:0_18:3) | GP | down |
| **HMDB0007883** | PC(14:0_20:4) | GP | down |
| **HMDB0007892** | PC(14:0_22:6) | GP | down |
| **HMDB0007949** | PC(15:0_20:4) | GP | down |
| **HMDB0007992** | PC(15:0_16:1) | GP | down |
| **HMDB0008023** | PC(16:1_22:6) | GP | down |
| **HMDB0008143** | PC(18:2_20:0) | GP | down |
| **HMDB0008156** | PC(20:4_20:4) | GP | down |
| **HMDB0008288** | PC(20:0_22:6) | GP | down |
| **HMDB0008596** | PC(18:2_22:1) | GP | down |
| **HMDB0008690** | PC(22:5_14:1) | GP | down |
| **HMDB0008970** | PE(16:1_20:4) | GP | down |
| **HMDB0009047** | PE(18:1_24:1) | GP | down |
| **HMDB0009382** | PE(20:4_14:0) | GP | down |
| **HMDB0009762** | PE(24:1_20:4) | GP | down |
| **HMDB0009788** | PI(16:0_20:3) | GP | down |
| **HMDB0009789** | PI(16:0_20:4) | GP | down |
| **HMDB0009846** | PI(18:2_16:0) | GP | down |
| **HMDB0009846** | PI(18:2_16:1) | GP | down |
| **HMDB0009881** | PI(20:3_18:1) | GP | down |
| **HMDB0009895** | PI(20:4_18:1) | GP | down |
| **HMDB0010393** | LPC(20:3/0:0) | GP | down |
| **HMDB0011708** | TG(15:0_18:1_18:2) | GL | down |
| **HMDB0011711** | TG(15:0_18:2_18:2) | GL | down |
| **HMDB0042143** | TG(14:0_16:0_22:5) | GL | down |
| **HMDB0042155** | TG(14:0_18:0_20:0) | GL | down |
| **HMDB0044077** | TG(16:0_16:1_20:5) | GL | down |
| **HMDB0044093** | TG(16:0_18:1_24:1) | GL | down |
| **HMDB0044096** | TG(16:0_18:1_20:2) | GL | down |
| **HMDB0044112** | TG(16:0_18:1_22:0) | GL | down |
| **HMDB0044120** | TG(16:0_18:1_22:1) | GL | down |
| **HMDB0044134** | TG(16:0_18:1_22:5) | GL | down |
| **HMDB0044263** | TG(16:0_18:2_18:3) | GL | down |
| **HMDB0044271** | TG(16:0_18:2_18:4) | GL | down |
| **HMDB0048638** | TG(16:1_18:1_22:1) | GL | down |
| **HMDB0048648** | TG(16:1_18:1_18:3) | GL | down |
| **HMDB0062701** | TG(17:0_18:1_18:1) | GL | down |

# Table S3 Identification results of differential metabolites of “GA vs. Mod”

| **HMDB** | **Compounds** | **Class.I** | **type** |
| --- | --- | --- | --- |
| **HMDB0000062** | DL-Carnitine | FA | up |
| **HMDB0000201** | Carnitine C2:0 | FA | up |
| **HMDB0000848** | Carnitine C18:0 | FA | up |
| **HMDB0002013** | Carnitine C4:0 | FA | up |
| **HMDB0002095** | Carnitine C3:1-2OH | FA | up |
| **HMDB0004948** | Cer(d18:1/18:1) | SL | up |
| **HMDB0004972** | HexCer(d18:1/18:0) | SL | up |
| **HMDB0007176** | DG(18:0_22:4) | GL | up |
| **HMDB0010203** | 14(S)-HDHA | FA | up |
| **HMDB0010203** | PGF2α | FA | up |
| **HMDB0010203** | TxB3 | FA | up |
| **HMDB0012087** | SM(d18:0/18:0) | SL | up |
| **HMDB0013130** | Carnitine C6-OH | FA | up |
| **HMDB0013326** | Carnitine C12:1 | FA | up |
| **HMDB0013330** | Carnitine C14:1-OH | FA | up |
| **HMDB0000138** | Glycocholic acid | FA | down |
| **HMDB0002580** | Taurolithocholic acid-3-sulfate | FA | down |
| **HMDB0007112** | DG(16:0_20:4) | GL | down |
| **HMDB0008004** | PC(16:1_18:1) | GP | down |
| **HMDB0008979** | PE(16:1_22:6) | GP | down |
| **HMDB0009041** | PE(18:1_22:2) | GP | down |
| **HMDB0009452** | PE(20:5_16:1) | GP | down |
| **HMDB0009880** | PI(20:3_18:0) | GP | down |
| **HMDB0009895** | PI(20:4_18:1) | GP | down |
| **HMDB0011474** | LPE(16:1/0:0) | GP | down |
| **HMDB0011506** | LPE(18:1/0:0) | GP | down |
| **HMDB0011512** | LPE(20:1/0:0) | GP | down |
| **HMDB0011513** | LPE(20:2/0:0) | GP | down |
| **HMDB0112417** | PS(18:1_22:1) | GP | down |

## Supplementary Figure

**
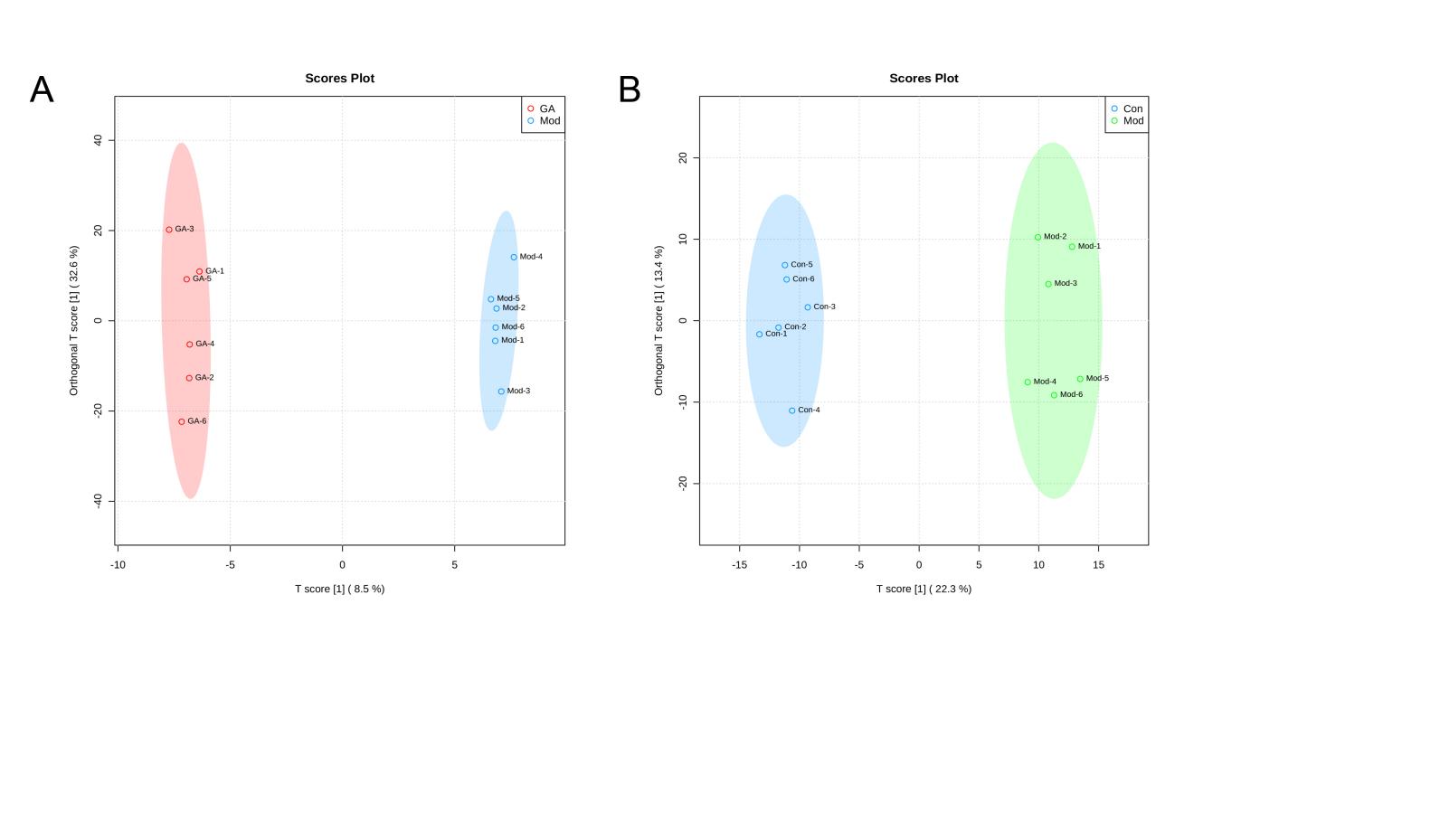
**

Figure S1. Metabolomics profiling of liver tissues from Con and Mod groups. (A) OPLS-DA plots of “Mod vs. Con”; (B) OPLS-DA plots of “GA vs. Mod”.
